# Changes in the Cardiotoxic Effects of Lead Intoxication in Rats Induced by Muscular Exercise

**DOI:** 10.3390/ijms23084417

**Published:** 2022-04-16

**Authors:** Svetlana V. Klinova, Ilzira A. Minigalieva, Yuri L. Protsenko, Marina P. Sutunkova, Vladimir B. Gurvich, Julia V. Ryabova, Irene E. Valamina, Oksana P. Gerzen, Salavat R. Nabiev, Alexander A. Balakin, Oleg N. Lookin, Ruslan V. Lisin, Daniil A. Kuznetsov, Larisa I. Privalova, Vladimir G. Panov, Leonid B. Katsnelson, Larisa V. Nikitina, Boris A. Katsnelson

**Affiliations:** 1 Yekaterinburg Medical Research Center for Prophylaxis and Health Protection in Industrial Workers, 620014 Yekaterinburg, Russia; klinovasv@ymrc.ru (S.V.K.); ilzira-minigalieva@yandex.ru (I.A.M.); marinasutunkova@yandex.ru (M.P.S.); gurvich@ymrc.ru (V.B.G.); ryabova@ymrc.ru (J.V.R.); ivalamina@mail.ru (I.E.V.); privalovaLI@yahoo.com (L.I.P.); vpanov@ecko.uran.ru (V.G.P.); 2 Institute of Immunology and Physiology of the Ural Branch of the Russian Academy of Sciences, 620049 Yekaterinburg, Russia; y.protsenko@iip.uran.ru (Y.L.P.); o.p.gerzen@gmail.com (O.P.G.); salavatik2003@gmail.com (S.R.N.); balakin_a_a@mail.ru (A.A.B.); o.lookin@iip.uran.ru (O.N.L.); lisin.ruslan@gmail.com (R.V.L.); virus_x@mail.ru (D.A.K.); leonidkatsnelson51@gmail.com (L.B.K.); laranikita63@gmail.com (L.V.N.); 3 Institute of Industrial Ecology, The Urals Branch of the Russian Academy of Sciences, 620049 Ekaterinburg, Russia

**Keywords:** Pb, running, cardiovascular system, contractility, isolated heart muscles, isomyosins

## Abstract

Exposure to lead is associated with an increased risk of cardiovascular diseases. Outbred white male rats were injected with lead acetate intraperitoneally three times a week and/or were forced to run at a speed of 25 m/min for 10 min 5 days a week. We performed noninvasive recording of arterial pressure, electrocardiogram and breathing parameters, and assessed some biochemical characteristics. Electrophoresis in polyacrylamide gel was used to determine the ratio of myosin heavy chains. An in vitro motility assay was employed to measure the sliding velocity of regulated thin filaments on myosin. Isolated multicellular preparations of the right ventricle myocardium were used to study contractility in isometric and physiological modes of contraction. Exercise under lead intoxication normalized the level of calcium and activity of the angiotensin-converting enzyme in the blood serum, normalized the isoelectric line voltage and T-wave amplitude on the electrocardiogram, increased the level of creatine kinase-MB and reduced the inspiratory rate. Additionally, the maximum sliding velocity and the myosin heavy chain ratio were partly normalized. The effect of exercise under lead intoxication on myocardial contractility was found to be variable. In toto, muscular loading was found to attenuate the effects of lead intoxication, as judged by the indicators of the cardiovascular system.

## 1. Introduction

Occupational exposure of an organism to harmful substances (particularly in mining and metallurgy) is associated, as a rule, with muscular work of greater or lesser intensity. The effect of this combination on the development of pathological processes has been studied, but research is insufficient. If an exposure is inhalational, then the absorbed dose is bound to be increased as a result of more intensive lung ventilation; however, one may expect that the extent of the organism’s response to unit dose may vary in either direction, due to the operation of different mechanisms associated with muscular work. For instance, it was demonstrated that, in rats with a developing experimental silicosis, this process may be both enhanced and attenuated when animals are forced to run every day, depending on the running speed [1,2].

Besides mining and metallurgy, the sources of environmental contamination with lead are lead–acid storage batteries, paints, ceramics and other household items [3,4]. Despite the reducing contamination of the environment with lead in recent decades [4,5], the problem of chronic intoxication with this element is still important. It has been shown in a number of experimental studies that cardiovascular risks under exposure to lead are increasing [6,7,8,9]. According to the Institute for Health Metrics and Evaluation [10], in 2017, lead exposure accounted for 1.06 million deaths. Moreover, exposure to this element was responsible for 10.3% of the global burden of hypertensive heart disease, 5.6% of the global burden of the ischemic heart disease and 6.2% of the global burden of stroke [10].

It has been found that the muscular training exercise may enhance the resistance of the athlete’s body to lead and some other metal-induced intoxications [11,12]. Similarly, Roshan et al. [13] and Shahandeh et al. [14] demonstrated that regular treadmill exercise may considerably attenuate neurotoxicity and reduce oxidative stress in rats exposed to lead acetate, while carrying out the running exercise to exhaustion was not associated with these beneficial effects.

The objective of this work was to study the effect of a mild running exercise on the condition of the cardiovascular system in rats under subchronic lead intoxication of a moderate severity.

## 2. Results

### 2.1. Development of Lead Intoxication and the Effect of Muscle Exercise on It

The statistically significant adverse shifts compared with the control were observed in 33.5% of the indices reflecting the status of the rat’s body. It is important to note that they included such lead-specific shifts as increased coproporphyrin in urine (614.46 ± 77.83 nmol/L in the “Pb” group against 184.30 ± 58.07 nmol/L in the control group; *p* < 0.05 by Student’s *t*-test), increased the percentage ratio of reticulocytes in peripheral blood (68.89 ± 4.90‰ in the “Pb” group against 21.75 ± 2.25‰ in the control group; *p* < 0.05 by Student’s *t*-test), and reduced the average hemoglobin content in the erythrocyte (14.96 ± 0.29 × 10^−12^ g in the “Pb” group against 20.78 ± 0.29 × 10^−12^ g in the control group; *p* < 0.05 by Student’s *t*-test).

The running parameters set on the treadmill were tolerated by the animals without any evident trouble, which enabled us to consider this running as a model of moderate muscular loading. Its effect on the development of lead intoxication was found to be varied: in 23.7% of the indices, the harmful effect of lead was enhanced; in 28.9% of the indices, it was attenuated; and in 47.4% of the indices, it remained unchanged.

### 2.2. Biochemical and Systemic Physiological Indices Associated with the Condition of the Heart, Blood Vessels and Breathing Function

The investigations revealed a reduced level of calcium in the blood serum in the “Pb” and “Running” groups (Table 1). Under the combined impact of lead and running, the level of calcium was somewhat closer to the control value as compared with results obtained under impact of lead only. It should be acknowledged, however, that this seemingly beneficial effect of the muscular exercise was only marginal. We observed an increase in creatine kinase-MB (CK-MB) in the “Pb + Running” group, which may indirectly point to an enhanced damaging effect of lead on the myocardium under physical exercise. Running exercise led to an increase in the vascular endothelial growth factor (VEGF) under lead intoxication, to an even greater extent than running alone.

The concentration of the angiotensin-converting enzyme (ACE) was found to increase under lead intoxication (Table 1). At the same time, we did not find any reliable changes in arterial pressure parameters.

The cardiomyocyte thickness of the lead-exposed group (5.38 ± 0.12 µm) was found to be higher than that of the control one (4.74 ± 0.08 µm); although this difference was relatively small, it was still statistically significant (*p* < 0.01). In the exercised group, the cardiomyocyte thickness was similarly increased (5.17 ± 0.09 µm) in comparison with the control one. In the “Pb + Running” group, the cardiomyocyte thickness (5.38 ± 0.10 µm) was also statistically significantly higher as compared with the control.

The electrocardiogram (Table 2) revealed a lowering of the isoelectric line in the second lead, which was partly offset under exercise. Additionally, lead intoxication was found to increase the amplitude of the T wave, with no such increase under exercise.

Lead intoxication increased the end-expiratory pause (Table 2). Exercise by itself did not affect this index. In the “Running” group, the breathing rate was increased, and the respiratory cycle was shortened due to a decrease in inspiratory time and end-inspiratory pause. It is interesting that the maximum inspiratory rate under lead intoxication tended to slow down in the “Pb” group and was reduced statistically significantly in the “Pb + Running” group. Additionally, under the combined impact, the relaxation time was shorter, i.e., the respiratory cycle under lead intoxication slowed down, while exercise shortened it. The combined action increased inspiratory time, while the relaxation phase became shorter; as a result, the duration of the cycle practically did not change.

### 2.3. Contractility of Isolated Myocardial Preparations

**Figure 1:**
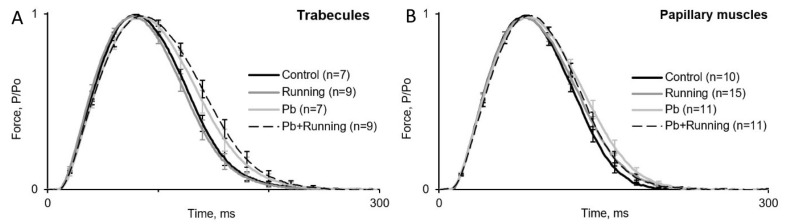
Superposition of the force development time course for steady-state isometric contractions of right ventricular trabecules (**A**) and papillary muscles (**B**) from male rats of the control group and the three exposed groups (here and in the following figures, designations are given in the legend, and the number of preparations is given in brackets). Force is given in units normalized to amplitude. Muscle length is 95% *Lmax*.

Figure 1 shows averaged isometric contraction curves for the trabecules (Figure 1A) and papillary muscles (Figure 1B) from the control and the three exposed groups, normalized to their amplitudes to enable comparison.

**Figure 2:**
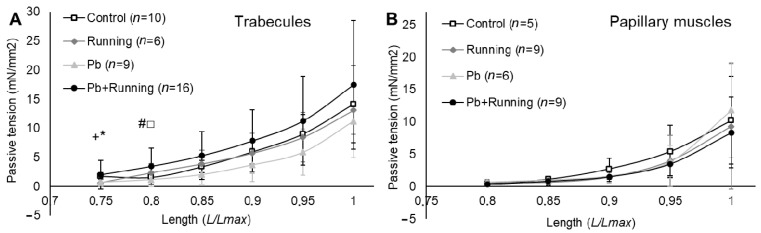
The relationships between passive tension amplitude and relative muscle length of trabecules (**A**) and papillary muscles (**B**) in the experimental groups. The number of preparations is given in brackets. Muscle length is shown in *Lmax* units. Data are shown as mean ± S.D. There are significant differences (by Mann–Whitney U-test): *—“Running” vs. “Control”; +—“Pb” vs. “Control”; #—“Pb + Running” vs. “Control”; □—“Pb + Running” vs. “Pb”.

The tendency towards passive tension (myocardial stiffness) reduction in the trabecules under lead intoxication was offset by exercise (Figure 2A). Running by itself did not influence this stiffness, while a combination of lead intoxication and running exercise increased trabecules stiffness. The respective curves for papillary muscles are seen to be located close to each other (Figure 2B).

**Figure 3:**
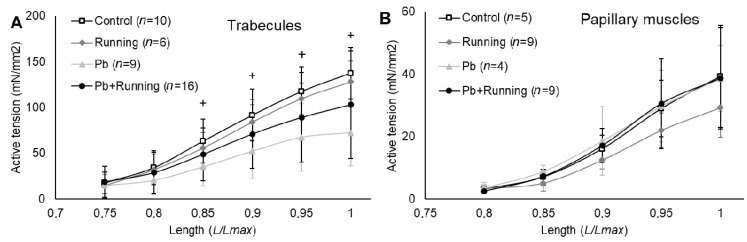
The relationships between active tension amplitude and relative muscle length of trabecules (**A**) and papillary muscles (**B**) in the experimental groups. The number of preparations is given in brackets. Muscle length is shown in *Lmax* units. Data are presented as mean ± S.D. There are significant differences (by Mann–Whitney U-test): +—“Pb” vs. “Control”.

It should be emphasized that we did not find an elevated arterial pressure under lead intoxication despite the increased ACE activity. At tissue level, we observed a negative inotropic effect of lead in the trabecules as a reduced active tension (Figure 3). The negative inotropic effect of lead intoxication was partly offset by running exercise (Figure 3).

**Figure 4:**
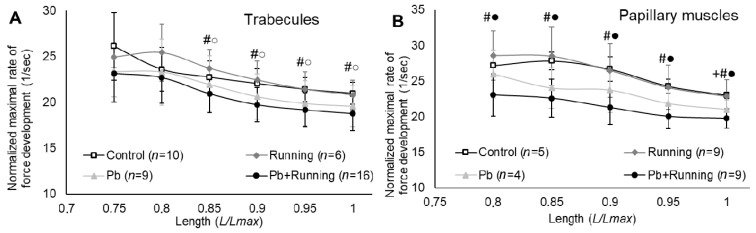
The relationships between maximal normalized rate and relative muscle length of trabecules (**A**) and papillary muscles (**B**) in the experimental groups. The number of preparations is given in brackets. Muscle length is shown in *Lmax* units. Data are presented as mean ± S.D. There are significant differences (by Mann–Whitney U-test): #—Pb + “Running“ vs. “Control”; ●—“Pb + Running” vs. “Running”; ○—“Pb” vs. “Running”.

The normalized maximal rate of tension development in the isometric mode of contraction (Figure 4.) decreased under a combined action of lead and physical exercise in both types of muscle. Physical loading appears to exacerbate the effect of lead in relation to this index.

**Figure 5:**
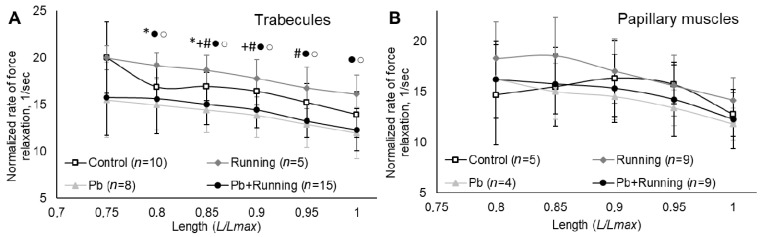
The relationships between normalized rate of force relaxation and relative muscle length of trabecules (**A**) and papillary muscles (**B**) in the experimental groups. The number of preparations is given in brackets. Muscle length is shown in *Lmax* units. Data are presented as mean ± S.D. There are significant differences (by Mann–Whitney U-test): *—“Running” vs. “Control”; +—“Pb” vs. “Control”; #—“Pb + Running” vs. “Control”; ●—“Pb + Running” vs. “Running”; ○—“Pb” vs. “Running”.

The normalized relaxation rate is reduced (Figure 5) under the impact of lead both by itself and in combination with exercise (statistically significantly in trabecules). Exercise alone increases this index somewhat.

**Figure 6:**
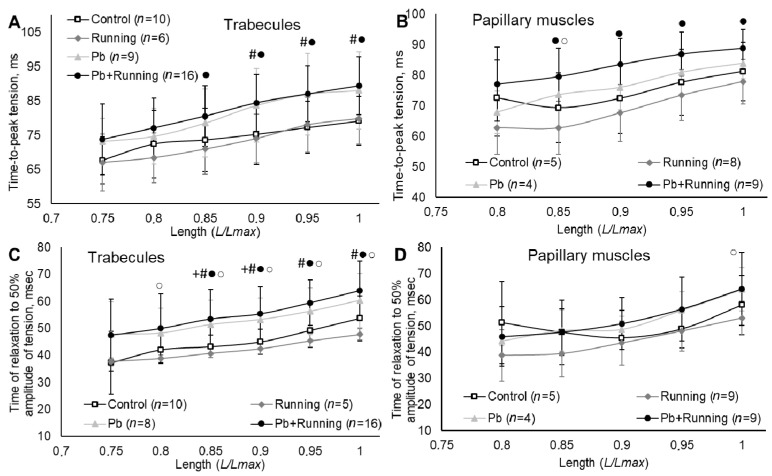
Isometric contraction time parameters of trabecules (**A**,**C**) and papillary muscles (**B**,**D**) in the experimental groups. The number of preparations is given in brackets. Muscle length is shown in *Lmax* units. Data are presented as mean ± S.D. There are significant differences (by Mann–Whitney U-test): +—“Pb” vs. “Control”; #—“Pb + Running” vs. “Control”; ●—“Pb + Running” vs. “Running”; ○—“Pb” vs. “Running”.

We observed growth in the contraction and relaxation (t50) times of the myocardial preparations (statistically significant in those of trabecules) from rats subjected to lead intoxication irrespective of running exercise (Figure 6). Physical loading by itself just slightly reduced the myocardial contraction and relaxation times.

**Figure 7:**
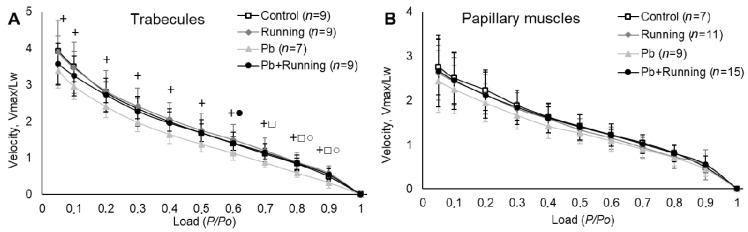
The force–velocity relationships of trabecules (**A**) and papillary muscles (**B**) in the experimental groups. Velocity is shown in relative units according to a muscle’s working length. The number of preparations is given in brackets. Data are presented as mean ± S.D. There are significant differences (by Mann–Whitney U-test): +—“Pb” vs. “Control”; ●—“Pb + Running” vs. “Running”; □—“Pb + Running” vs. “Pb”; ○—“Pb” vs. “Running”.

Exposure to lead reduced the maximal rate of isotonic shortening in trabecules (Figure 7A). The curves for papillary muscles are seen to be close to each other (Figure 7B); although, here as well, we can see a statistically insignificant decrease in the maximal rate of isotonic shortening under exposure to lead. Exercise normalized this indicator in both types of cardiac muscle preparations.

**Figure 8:**
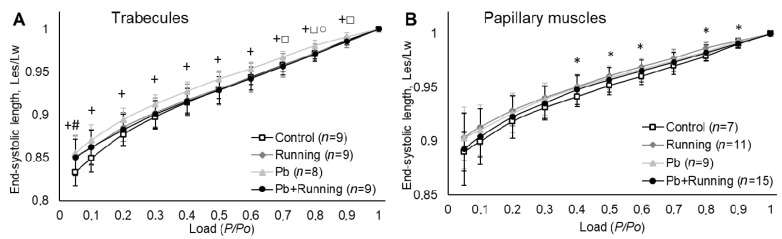
The end-systolic length–afterload relationships of trabecules (**A**) and papillary muscles (**B**) in the experimental groups. The number of preparations is given in brackets. Data are presented as mean ± S.D. There are significant differences (by Mann–Whitney U-test): *—“Running” vs. “Control”; +— “Pb” vs. “Control”; #—“Pb + Running” vs. “Control”; □—“Pb + Running” vs. “Pb”; ○—“Pb” vs. “Running”.

The end-systolic length is seen to grow (Figure 8) under exposure to lead in trabecules and under exercise in papillary muscles.

**Figure 9:**
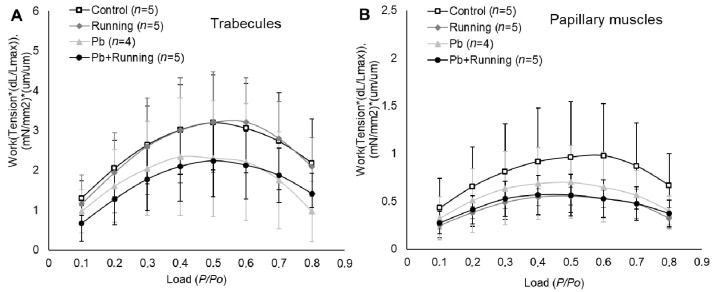
The work–afterload relationships of trabecules (**A**) and papillary muscles (**B**) in the experimental groups. The number of preparations is given in brackets. Data are presented as mean ± S.D.

Mechanical work produced by cardiac muscle preparations (myocardial efficiency) (Figure 9) had no statistically significant differences for any experimental groups. There was a tendency towards reduced myocardial efficiency under exposure to lead.

### 2.4. Myosins

The maximal velocity of reconstituted thin filament sliding over rat myosin (Vmax) under lead intoxication was reduced significantly (Table 3). Under exercise, the Vmax of experimental rats was not different from that in the control group. However, as a result of running exercise under lead intoxication, Vmax was increased compared with the “Pb” group but did not reach the “Control” and “Running” group values, i.e., with the running exercise, the effect of lead intoxication was attenuated as per the index Vmax.

**Figure 10:**
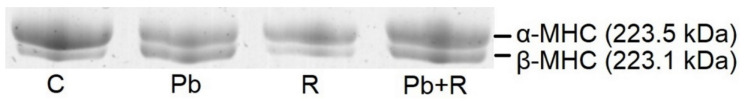
Representative examples of electropherograms showing the ratio of α- and β-MHC extracted from the RV rat myocardium. Left to right: “Control”; “Pb”; “Running”; “Pb + Running”.

All changes in the sliding velocity of reconstituted thin filament over myosin correlated with changes in the ratio of α- and β-myosin heavy chains (Table 3, Figure 10).

### 2.5. Analysis of Combined Action with the Help of RSM

When performing RSM analysis, we observed a complicated picture of combined action of lead and exercise on the indices of the cardiovascular system.

**Figure 11:**
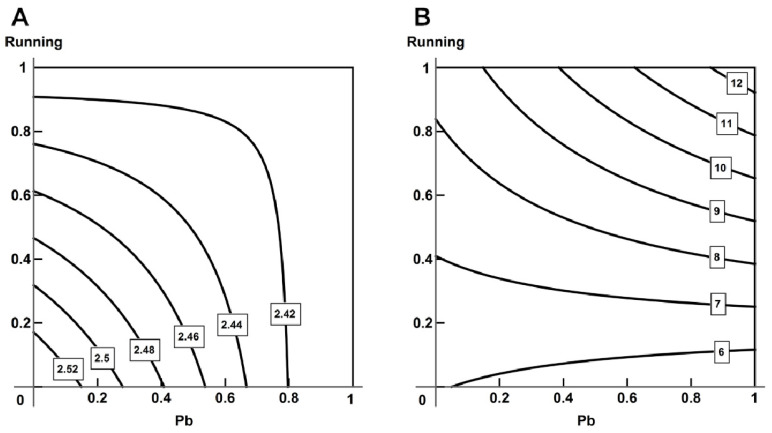
Variability of combined action of running and lead intoxication on (**A**) total Ca (from additive action towards subadditivity) and (**B**) VEGF (from single-factor action of exercise, through synergism, to additivity) in blood serum. Regression equations are $y=2.543-0.155x_{1}-0.136x_{2}-0.165x_{1}x_{2}$ for (**A**) and $y=6.046-0.912x_{1}+2.332x_{2}+5.120x_{1}x_{2}$ for (**B**).

Exercise reduced the toxic effect of lead judging by the level of calcium in the blood (Figure 11A), changing from combined additive action to subadditivity. In relation to other biochemical indices, we observed a complicated picture of combined action, in which the leading factor was exercise, but at higher lead concentrations we observed an additive action (Figure 11B).

**Figure 12:**
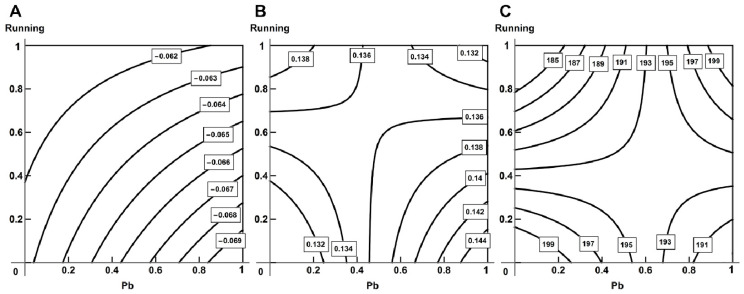
Variability of combined action of running and lead intoxication on (**A**) isoline amplitude (2 lead, opposite action), (**B**) amplitude T (2 lead, complicated picture: from subadditive action at low doses of lead and exercise, through antagonism, to synergism at high doses), (**C**) inspiratory time (complicated picture: same as in (**B**)). Regression equations are $y=-0.063-0.007x_{1}+0.002x_{2}+0.006x_{1}x_{2}$ for (**A**), $y=0.127+0.019x_{1}+0.013x_{2}-0.028x_{1}x_{2}$ for (**B**) and $y=202.7-14.283x_{1}-22.568x_{2}+35.570x_{1}x_{2}$ for (**C**).

The impact of lead was attenuated by exercise, judged, for instance, by the duration of the QT interval and isoelectric line voltage in the second lead (oppositely directed action, Figure 12A). Some electrocardiographic indices, too, presented a complex picture of combined action (for instance, the T-wave amplitude in the second lead, Figure 12B).

The majority of hemodynamic indices and breathing parameters revealed oppositely directed actions of lead and running; although, some indices presented a complicated picture of combined action (Figure 12C).

**Figure 13:**
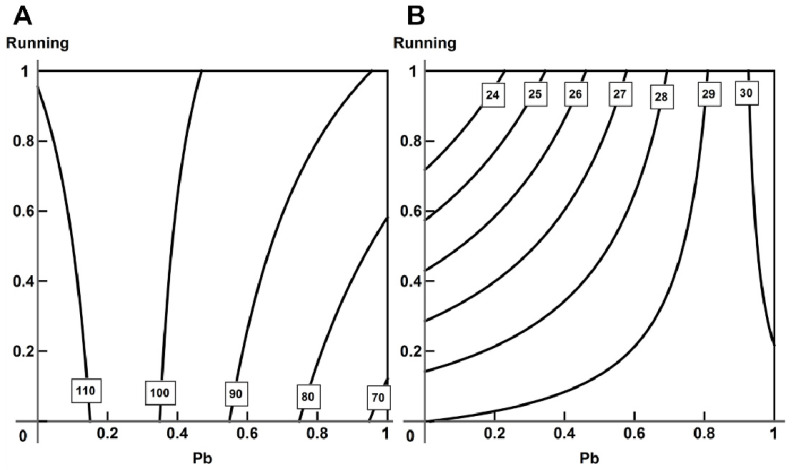
Variability of combined action of running and lead intoxication on active tension in (**A**) trabecules (single-factor effect of lead at low doses changes to opposite action at high doses) and (**B**) papillary muscles (opposite action at high doses of lead changes to its single-factor action at high doses) at L = 0.95 *Lmax*. Regression equations are $y=117.5-50.120x_{1}-7.864x_{2}+29.557x_{1}x_{2}$ for (**A**), and $y=28.987+0.836x_{1}-6.953x_{2}+7.763x_{1}x_{2}$ for (**B**).

The effect of lead intoxication on active tension is attenuated by exercise: we observed transition from a single-factor action of lead at its low concentrations to an oppositely directed action at high concentrations (Figure 13A). On the contrary, in papillary muscles we saw a contra-directional action, which changed to a single-factor action of lead at L = 0.95–1.0 *Lmax* and high doses of lead (Figure 13B).

**Figure 14:**
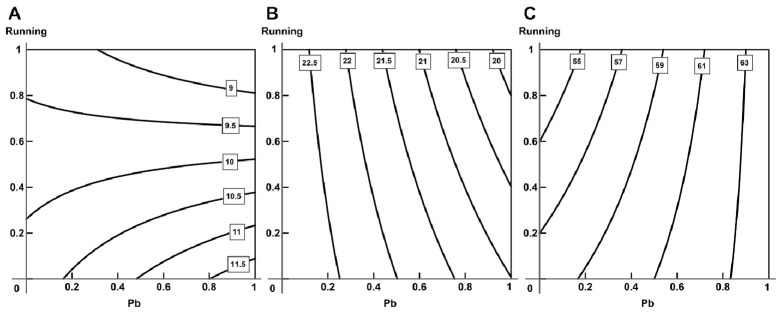
Variability of combined action of running and lead intoxication on (**A**) passive tension (single-factor action of running at low doses of lead changes to opposite and additive at high doses), (**B**) maximal normalized rate (single-factor action of lead at low doses changes to additive at high doses), (**C**) time of relaxation to 50% amplitude of tension (opposite action of lead at low doses changes to single-factor action at high doses) for L = 1.0 *Lmax* in papillary muscles. Regression equations are $y=10.248+1.562x_{1}-0.951x_{2}-2.516x_{1}x_{2}$ for (**A**), $y=23.002-1.990x_{1}-0.135x_{2}-1.130x_{1}x_{2}$ for (**B**) and $y=58.0+5.60x_{1}-5.0x_{2}+5.111x_{1}x_{2}$ for (**C**).

As for passive tension, the leading contribution of running reduced with increasing the dose of lead. For all Ls analyzed, we observed additivity or antagonism (subadditivity or opposite actions of the factors) at high “doses” of lead and exercise; although, at medium doses of running, its action prevailed (Figure 14A).

Physical loading attenuated the effect of lead intoxication on the rate of isometric contraction. We observed additivity under a combination of high “doses” of lead and exercise in relation to tension development rate (Figure 14B) and an opposite action in relation to relaxation rate.

As for time, the leading factor is lead intoxication—it prevailed over the single-factor action of lead (Figure 14C).

Running attenuated the toxic effect of lead on isotonic shortening rate. In trabecules, we observed an opposite action of the factors; in papillary muscles, we observed a complicated picture, which changed from a single-factor action of lead to a single-factor action of running.

Reduction in work produced by muscles under lead intoxication decreased with increasing exercise: in trabecules, it changed from a single-factor action of lead to a contra-directional one; in papillary muscles, it changed from subadditivity to a single-factor effect of running.

In general, the isobole analysis did not reveal indices in which exercise would explicitly enhance the effect of lead intoxication on the indices of the cardiovascular system; although, for some combinations of doses, producing complicated pictures of combined action, we observed synergism in some indices (for instance, Figure 11B and Figure 12B,C).

## 3. Discussion

Repeated parenteral (intraperitoneal in particular) injections of a toxic metal salt solution present a widely used approach to experimental modelling of the essential features and mechanisms of a corresponding chronic or subchronic metal-induced intoxication that are not associated with the responses that depend on the target for corresponding “natural” exposure (oral, inhalational or transcutaneous). The accuracy of dosage and reliable reproducibility are the main advantages of this model in solving quantitative problems of a comparative character. On the contrary, it would be hardly possible to obtain on the rat an adequate model of real physical work of humans. Even if this work involves walking or running, it engages muscle groups other than those employed in the forced running of a four-legged animal. Thus, such running simulates just very general physiological responses of the organism to energy expenditure associated with increased muscle activity.

Although the dose of lead given to rats in our experiment may seem very high as compared with typical human exposures (see, e.g., [15]), intoxication that developed in the lead-only group may be characterized as moderate. The changes in coproporphyrin, percentage ratio of reticulocytes and hemoglobin content in the erythrocyte point to typical disturbances to porphyrin metabolism and heme synthesis under lead intoxication.

In the electrocardiogram (Table 2), the lowering under lead intoxication of the isoelectric line may point to toxic damage to the myocardium and metabolic disturbances in it. The growth in the T-wave amplitude at the same group points to an impairment of myocardial repolarization processes, which may influence the myocardial relaxation phase in the cardiac cycle.

In the case of lead intoxication, a reduced level of serum calcium (Table 1) is associated with lead–calcium antagonism [16,17], while under physical loads it may be tentatively explained by elevated demand of muscles for calcium [18,19].

VEGF ensures stimulation of proliferation and growth of vascular endothelial cells and enhances their viability [20]. It is known that lead can cause hypoxia in cells and tissues [21]. Thus, an increase in the VEGF in the “Pb + Running” group may be the organism’s compensatory mechanism against hypoxia.

Various researchers have shown association between growth in the blood lead concentration and development of hypertension (for instance, [22,23]). In our study, however, we did not find any reliable changes in arterial pressure parameters.

The absence of a significant reduction in work produced by muscles (Figure 9) in the physiological range of afterloads (P/Po = 0.3–0.7) is evidence of stability and strength of the heart’s protective mechanisms.

We observed growth in cardiomyocyte thickness and trabecules stiffness in the “Pb + Running” group (Figure 2). These impairments may be a precursor to myocardial hypertrophy since the latter itself contributes to increased stiffness [24].

In in vivo conditions, the sarcomeres of cardiomyocytes in the ventricular wall during the diastole are stretched to lengths exceeding 85% *Lmax* [25,26]; therefore, the measurements performed by us for lengths L ≥ 75% *Lmax* in trabecules and L ≥ 80% *Lmax* in papillary muscles may be interpreted as physiologically relevant. The increased stiffness of the myocardium at the sarcomere’s physiological lengths (1.6—2.2 mcm) in a stretched myocardium is largely due to the stretching of the titin protein (from 3000 to 3700 kDa) [27]. The relationship between myocardial stiffness and titin in the trabecules may vary under the effect of calcium ions [28,29]. The partial substitution of lead for calcium ions under lead intoxication found in experimental studies [30,31] may result in a reduced contribution of calcium to titin’s stiffness.

One of the important primary mechanisms underlying the toxic effect of lead is an oxidative stress, which may also influence the level of myocardial passive stiffness. Under oxidative stress, stiffness increases in some regions of titin’s gigantic molecules and reduces in others, depending on the structure of corresponding domains. The overall effect of these changes has not yet been studied in full [29].

The end-systolic length is seen to grow (Figure 8) under exposure to lead in trabecules and under exercise in papillary muscles, which may be partly associated with the reduced myocardial stiffness we found and provides indirect evidence of an increased volume of ejection in the heart (Figure 2).

Some difference in the results of lead’s effect on papillary muscles and on trabecules may be explained by the physiological adaptation of wall trabecules to relatively low loads in the natural cardiac cycle compared with higher loads on the papillary muscles, holding the tricuspid valve closed in the whole heart. Thus, we may regard as one of the mechanisms of heart’s adaptation to the impact of lead the reduced passive stiffness of just the trabecules under the impact of lead acetate since they pertain to the ventricular wall. It is probable that the ventricle’s “softer” wall, in the course of diastolic filling the sarcomeres, would be stretched a little longer than with a stiffer one, and the end-diastolic volume in this case would be increased. However, we found negative inotropic effect of lead in the trabecules (Figure 3). The reduced ability of the wall trabecules to generate force may be the reason for the absence of growth in systolic force in the whole heart, and it may correlate with the absence of changes in arterial pressure, contrary to the data obtained by other authors (e.g., [32,33]).

The myocardial recovery processes may be assessed by the relaxation phase of “contraction–relaxation” cycle in the muscles from control and experimental rats (Figure 5). In exercised rats, the recovery processes in their myocardium proceed faster while lead intoxication slows them down. The combined effect of these two factors is determined by the influence of lead.

The growth in the isometric contraction time that we found (Figure 6) is a sign of possible myocardial hypertrophy. Such data were obtained when modelling rat myocardium hypertrophy by ligation or under the impact of the pyrrolizidine alkaloid, monocrotaline, causing pulmonary artery endothelial hyperplasia [34].

A reduction in the rate (Figure 5) and growth in the time indicators (Figure 6) provide evidence of the “contraction–relaxation” cycle becoming longer under lead intoxication, which may serve as an adaptation (compensatory) mechanism for maintaining the ejection fraction. This peculiarity of lead’s impact on myocardial contractility was demonstrated by us previously [35,36]. The presence of physical loading under lead intoxication did not influence significantly the rate and time characteristics of isometric contractions compared with exposure to lead alone. Exercise by itself could produce an effect on the isometric contraction rate and time indicators of trabecules and papillary muscles, but it was opposite to that of lead.

The time course of tension development and relaxation in isometric contraction is determined by two main factors: myosin isoform ratio and rates of processes participating in the calcium regulation of contractions. Our data showing changes in the ratio of fast and slow myosin isoforms towards slower ones (Table 3, Figure 9) may be a precursor to myocardial hypertrophy, which for the given duration of the experiment involving intraperitoneal injections manifested itself at molecular level only. An additional argument in favor of the hypertrophy suggestion is the fact that, in this experiment and in a similar one, cardiomyocytes in histological preparations were shown to become thicker under lead intoxication [37].

Under lead intoxication, the reduction in isometric contraction rates and in the maximal rate of isotonic shortening correlates with a reduction in the sliding velocity of regulated thin filaments and changes in the ratio of α- and β-MHC, and is partly explained by growth in the proportion of slower β-MHC. These correlations were shown by us previously in similar experiments [38,39].

Exercise in the form of running, as used in the experiment, did not cause changes in velocity and the ratio of α- and β-MHC. As a consequence, on the tissue level of the myocardial structure, we did not observe any changes in the contraction rates of multicellular preparations in either isometric or the physiological mode.

At the same time, under combined action of lead and exercise, we observed an attenuated action of lead manifesting itself in the normalization of both the sliding velocity of regulated thin filaments and the ratio of α- and β-MHC, which led, at tissue level, to the normalization of the maximal rate of isotonic shortening. However, the maximal rate of isometric contraction development remained reduced. The rate characteristics of tension development in the isometric mode and shortening in the isotonic mode depend on two main factors: the kinetics of myosin cross-bridges determined by the ratio of α- and β-MHC and rates of calcium handling in the cardiomyocyte.

These findings suggest that in a myocardium exposed to a combined action of lead and muscular loading in isotonic conditions, the decisive factor is the recovery of the kinetics of myosin cross-bridges under the effect of exercise. At the same time, the development of isometric tension is largely controlled by calcium handling, which seems to have remained unchanged in response to muscular loading.

## 4. Materials and Methods

### 4.1. Experimental Animals, Exposures and Toxicological Indices

The study was performed on outbred male rats from our own breeding colony. The body mass of the animals as at the start of the study was around 250 g and the age was 3.5–4 months. The rats were kept under standard conditions in a normal atmosphere, and ate balanced food for rodents. In planning and conducting the experiments, we followed the “International Guiding Principles for Biomedical Research Involving Animals” of the Council for International Organizations of Medical Sciences and the International Council for Laboratory Animal Science (2012). Approval was obtained from the Ethics Committee of the Yekaterinburg Medical Research Center for Prophylaxis and Health Protection in Industrial Workers.

Between 10 and 15 rats were randomly selected for each of the 4 experimental groups. The first group of animals was exposed to lead acetate (“Pb”); the second was exposed to running exercise (“Running”); the third was exposed to these two agents together (“Pb + Running”); and the fourth group was the control (“Control”).

The rats in the corresponding groups were intraperitoneally injected with a solution of lead acetate trihydrate over 6 weeks, 3 times a week, at a dose of 11 mg/kg body mass. Such a dose had been previously chosen in several tentative experiments. The animals in the groups “Running” and “Control” received 2 mL of normal saline.

Exercise was modeled on a treadmill for rats, “TSE Treadmill System GmbH” (“TSE Systems International Group”, Germany). Before starting the experiment, the rats were trained to run every day for 5 min during 5 days with the foot shock option turned off, at a speed of 8 m/min. Then, over 3 weeks, the running workload was increased from 8 to 22 m/min (at a step of 3 m/min) for 10 min/day, 5 days/week. Then, in parallel to injections, the animals ran for 10 min/day 5 days a week at a speed of 25 m/min over 6 weeks.

During the 5th week of intraperitoneal injections, we performed noninvasive recording of electrocardiograms, breathing parameters (using an ecgTUNNEL system (emka TECHNOLOGIES, Paris, France)) and arterial pressure (with the help of a CODA-HT8 system (Kent Scientific, Torrington, CT, USA)).

Upon completion of the experiment, we gathered body blood samples for determination of hematological indices, including hemoglobin level, by a “Methic 18” hematological analyzer. Reticulocytes were counted in routine manner. The following indices were assessed in the blood serum: calcium, vascular endothelial growth factor (VEGF), endotelin-1, concentration of angiotensin-converting enzyme (ACE) and creatine kinase-MB (CK-MB). Daily urine was used to determine coproporphyrin contents biochemically. Histological changes in the myocardium were assessed morphometrically by measuring the cardiomyocytes’ thickness.

### 4.2. Determination of α– and β–Cardiac Myosin Heavy Chain (MHC) Ratio

Denaturing gel electrophoresis (SDS–PAGE) was employed to determine the myosin heavy chain isoform ratio in the myocardial tissue. After the electrophoresis, the gels were subjected to Coumassie Blue staining, then destained with a solvent and water and scanned in a GS-800 Calibrated Densitometer (BioRad, Hercules, CA, USA). The α– and β–MHC ratio was determined in the samples as percentage by Image Lab 5.2.1.

### 4.3. Assessment of the Mechanical Characteristics of Actin–Myosin Interaction by In Vitro Motility Assay

The mechanical characteristics of actin-myosin interaction were assessed by observing the movement of a fluorescently labeled regulated thin filament (including actin, tropomyosin and troponin) in a flow cell in the presence of ATP and calcium ions (pCa = 4). The rat myosin in the flow cell and the trabecules and papillary muscles were all isolated from the same hearts. We obtained actin from rabbit skeletal muscle according to standard procedure [40]; and myocardial troponin from a porcine left ventricle according to [41]. Recombinant tropomyosin was derived as described by Matyushenko et al. [42]. It is usual practice to use contractile and regulatory proteins extracted from animals of different species and combined in a motility assay [43,44]. Thin filaments were obtained by mixing actin, troponin and tropomyosin as follows: 400 nM rhodamine–phalloidin labeled F–actin, 100 nM troponin, and 100 nM tropomyosin at 4 °C in the buffer (25 mM KCl, 25 mM imidazole, 4 mM MgCl_2_, 1 mM EGTA, and 10 mM DTT, pH 7.5). The thin filaments’ protein ratio was verified by 10% SDS–PAGE [45].

The in vitro motility assay technique followed the procedure described in [46]. The temperature across all experiments was 32 °C. Thin filaments were fluorescently labeled and visualized using an Axiovert 200 inverted epifluorescence microscope with a 100×/1.45 Oil alpha Plan–Fluar objective (Carl Zeiss Microscopy, LLC, White Plains, NY, USA) and an EMCCD iXon–897BV camera (Oxford Instuments-Andor, Oxford, MS, USA). In each flow cell, we recorded ten 30 sec fields. Data analysis was performed using GMimPro software [47] with the velocities of >100 individual filaments averaged to obtain the mean value.

### 4.4. Measuring the Contractile Response of Isolated Myocardial Preparations

The rats representing all groups were given heparin (1000 ME, 0.25 mL per animal) prior to killing them by cervical dislocation. Upon euthanasia, the heart was immediately replaced and maintained in Krebs–Henseleit solution containing 2.3–butanedione monoxime (30 mM) for a quarter of an hour. The right ventricle of one and the same heart was taken to make preparations of trabecular and pupillary muscles. These were then attached to the rods of a length servomotor and force transducer in a temperature-controlled bath (Muscle Research System, Scientific Instruments GmbH, Gilching, Germany).

Experiments were carried out in a modified Krebs–Henseleit solution (in mM: NaCl 118.5; NaHCO_3_ 14.5; KCl 4.2; KH_2_PO_4_ 1.2; MgSO_4_ 1.2; glucose 11.1, CaCl_2_ 1.9), and oxygenated by a mixture of 95% O_2_ and 5% CO_2_, pH = 7.4 at 35 °C. This solution was circulated through the temperature-controlled bath (5 mL byy volume) by a peristaltic pump at a speed of 5 mL/min. Non-polarizable carbon electrodes were used to pace the trabeculae and papillary muscle preparations at 2 Hz by ~5 ms super-threshold rectangular stimuli. Measurements were performed at a temperature of 35 °C for a working length of 0.95 *Lmax*.

The mechanical response was measured under an isometric or physiological mode of contraction by an analogue–digital and digital–analog converter (PCI–1716S, AdLink Technology Inc., Taoyuan City, Taiwan) at a frequency of 10 kHz. The physiological mode involved a sequence of loads applied to the muscle similar to the physiological sequence in the cardiac cycle, enabling us to measure force–velocity relationships under different afterloads and force-shortening loops (which resemble the pressure–volume loop typical of the whole heart) [38,39,48,49]. In isometric conditions, the muscle length was increased incrementally to the muscle’s maximal active isometric force (*Lmax*), determined individually for each of the preparations. This value provided a reference point for contractility analysis. Since myocardial preparations from different rats differed in thickness, comparisons between them were enabled by normalizing the force developed by each muscle to its cross-sectional area calculated for the muscle’s central part from its larger and smaller diameters measured with a binocular stereoscopic microscope. The muscles were assumed to be ellipsoidal-shaped. Force was normalized to the cross-sectional area estimated as described above to determine mechanical tension. This approach differs from that of other researchers [30], who normalized active force to muscle weight. In this normalizing method, preparations of the same mass may have different diameters and, thus, the masses of a muscle’s parts that are employed for attaching it must be carefully determined and excluded from active force generation. We normalized the time course of isometric contraction to its amplitude to assess force development and relaxation rates.

### 4.5. Mathematical Processing and Analysis

We estimated the statistical significance of differences between the groups in the mean values of the indices by two-way factorial ANOVA with post hoc Tukey HSD test for functional and biochemical indices (multiple comparisons) or by Mann–Whitney U–test for mechanical characteristics (compared pairwise). (Based on similar results of our other experiments of this type, we believe that a typical distribution of these characteristics is not normal.) The Wolfram Research Mathematica v. 11.3 system was used for statistical treatment. The results are shown as mean ± s.e. Differences were considered statistically significant at *p* < 0.05.

Following our previous publications (e.g., [50,51]) and a recent study on magnetite nanocrystal clusters [52], we performed mathematical modelling using the response surface methodology (RSM) [53,54].

The regression equation describing the response surface *Y* = *Y* (*x*_1_, *x*_2_) is given by:

(1) $$Y=b_{0}+b_{1}x_{1}+b_{2}x_{2}+b_{12}x_{1}x_{2}$$

where *Y* is a toxicity index, and *x*_1_ and *x*_2_ are the doses of the agents participating in the combination. This equation was constructed by fitting the coefficients *b*_0_, *b*_1_, *b*_2_ and *b*_12_ to experimental data using the ordinary least squares method. Two agents are assumed to produce an effect in the same direction on response *Y* if both one-way response functions *Y*(*x*_1_, 0) and *Y*(0, *x*_2_) either increase or decrease with increasing *x*_1_ or *x*_2_; on the contrary, two agents are considered to be acting in opposite directions if one function increases while the other decreases. In the response surface methodology, even in the case of two-level agents, Equation (1) enables response y to be predicted for any combination of doses, even for two-level agents, within the experimental range for each of them rather than at two points only. Quasi-sectioning of the response surface on different levels corresponding to different meanings of outcome Y provides a family of Loewe isoboles, which may have the same or different shape and slopes in the same or opposite direction. It makes it easy to identify and demonstrate the type of binary combined toxicity.

A nonlinear polynomial model only could be used in an experiment if exposure levels are represented by common orthogonal encoding. In particular, no quadratic model is applicable to the data. Typically, the value of the determination coefficient R^2^ for Equation (1) was at least 0.5.

## 5. Conclusions

1. Under moderate subchronic intoxication with lead, we observed the following:

A shift of the myosin heavy chain ratio towards slower β-MHC, the growth in myocardial contraction time, a thickening of cardiomyocytes and a reduction in the sliding velocity of reconstituted thin filaments in an in vitro motility assay may point to the development of concentric cardiac hypertrophy. However, we can see only the point of the beginning of hypertrophy when there are changes at the molecular level, but there was no significant manifestation in contractility and structure of myocardium at the tissue level. There was an unaffected efficiency of the contractile apparatus, as assessed by the amount of work produced by isolated muscles; in the whole organism, this of great hemodynamic importance, and the lack of changes in it points to the operation of compensatory mechanisms in the heart in response to the impact of lead.

2. In the context of moderate lead intoxication in rats, the repeated short-term exercise applied in this experiment generally produced a positive effect on the cardiovascular system, as follows:

Physical training under lead intoxication normalized calcium level and the activity of the angiotensin-converting enzyme in the blood serum, isoelectric line voltage and T-wave amplitude in the electrocardiogram. At the same time, the level of creatine kinase-MB increased and the breathing indices decreased judging by the inspiratory rate and relaxation time.

Combination of exercise and lead intoxication partly normalized the maximum siding velocity of regulated thin filaments on myosin, which correlated with partial normalization of the myosin heavy chain ratio.

The effect of exercise under lead intoxication on myocardial contractility is variable: the active tension and maximum rate of isotonic shortening in trabecules were normalized but the maximum rate of force development in the isometric mode reduced in both types of muscle.

3. Using the response surface methodology, we confirmed the typological diversity of combined actions produced by lead and physical load on the cardiovascular system of rats, depending on the effect by which it is assessed, the ratio of doses and the type of object studied.

## Figures and Tables

**Table 1:** Some biochemical indices of rat’s blood serum in the experiment.

Indices	Groups of Rats Exposed			
	Normal Saline (Control)	Pb	Running	Pb + Running
Number of Rats	9	10	10	15
Total Ca, mmol/L	2.487 ± 0.042	2.353 ± 0.029 *	2.368 ± 0.031 *	2.388 ± 0.028
CK-MB, U/L	1337.13 ± 123.30	1358.82 ± 105.66	1398.46 ± 106.53	1750.23 ± 122.78 *#●
ACE, U/L	143.00 ± 14.74	236.36 ± 21.95 *	166.64 ± 13.44 #	135.91 ± 15.22 #
VEGF, 10^6^ U/mL	5.67 ± 0.40	5.13 ± 0.31	8.10 ± 0.74 *#	12.58 ± 1.68 *#●

Note: differences are statistically significantly for *p* < 0.05 by Tukey HSD test. *—from the “Control”; #—from the “Pb” group; ●—from the “Running” group.

**Table 2:** Electrocardiographic data.

Indices	Groups of Rats Exposed			
	Normal Saline (Control)	Pb	Running	Pb + Running
ECG 2nd Lead				
Number of Rats	10	10	10	10
RR interval, ms	156.87 ± 4.01	161.71 ± 2.88	148.62 ± 4.61 #	163.13 ± 5.77
Heart rate, bpm	378.52 ± 8.97	373.46 ± 6.77	408.45 ± 12.57 #	372.00 ± 13.04
Isoelectric line, mV	−0.06082 ± 0.00091	−0.0713 ± 0.0025 *	−0.0596 ± 0.0032 #	−0.0635 ± 0.0051
PQ interval, ms	41.76 ± 0.74	44.21 ± 0.93	44.24 ± 0.96	44.92 ± 1.14 *
QRS interval, ms	24.79 ± 0.45	25.43 ± 0.20	24.83 ± 0.73	24.66 ± 0.58
QT interval, ms	64.64 ± 1.78	66.89 ± 0.60	63.89 ± 1.80	64.86 ± 0.28 #
T amplitude, mV	0.1250 ± 0.0028	0.1506 ± 0.0074 *	0.138 ± 0.011	0.136 ± 0.011
Respiratory flow				
Number of rats	10	11	11	12
Respiratory cycle duration (peak to peak), ms	437.97 ± 15.48	459.79 ± 33.09	381.89 ± 9.01 *#	406.01 ± 16.12
Respiratory rate (peak to peak), bpm	138.51 ± 4.80	136.13 ± 8.85	157.83 ± 3.79 *#	150.14 ± 6.02
Inspiratory time, ms	200.38 ± 6.08	187.91 ± 3.98	177.81 ± 5.30 *	201.05 ± 6.86 ●
Peak inspiratory flow, ml/s	10.61 ± 1.17	7.96 ± 1.52	12.37 ± 1.57	7.95 ± 1.41 *●
Expiratory volume/inspiratory volume ratio	−1.41 ± 0.58	−0.63 ± 0.33	0.090 ± 0.138 *	−0.58 ± 0.32
End-inspiratory pause, ms	8.20 ± 0.50	7.24 ± 0.27	6.85 ± 0.14 *	7.13 ± 0.24
Relaxation time, ms	189.63 ± 11.42	174.07 ± 14.99	166.74 ± 7.76	158.73 ± 6.73 *
End-expiratory pause, ms	21.90 ± 0.31	23.96 ± 0.58 *	21.45 ± 0.39 #	23.51 ± 0.44 *●
penH	−0.356 ± 0.054	−0.65 ± 0.18	−0.311 ± 0.017	−0.503 ± 0.064 ●

Note: differences are statistically significantly for *p* < 0.05 by Tukey HSD test. *—from the “Control”; #—from the “Pb” group; ●—from the “Running” group.

**Table 3:** Maximal velocity of reconstituted thin filament sliding over RV myosin and the ratio of α- and β-myosin heavy chains in the right ventricle.

Indices	Groups of Rats Exposed			
	Normal Saline (Control)	Pb	Running	Pb + Running
Number of Rats	10	10	10	10
Vmax, µm/s	6.19 ± 0.16	4.70 ± 0.10 *	6.26 ± 0.17 #	5.77 ± 0.16 *#●
α-MHC, %	85 ± 5	53 ± 4 *	82 ± 3 #	68 ± 4 *#●
β-MHC, %	15 ± 5	47 ± 4 *	18 ± 3 #	32 ± 4 *#●

Note: differences are statistically significant for *p* < 0.05 by Mann–Whitney U-test: *—from the control, #—from the “Pb” group, ●—from the “Running” group.

## Data Availability

The data presented in this study are available on request from the corresponding author.
